# The association between the suicide crisis syndrome and suicidal behaviors: The moderating role of personality traits

**DOI:** 10.1192/j.eurpsy.2021.2235

**Published:** 2021-10-13

**Authors:** Jada Flint, Lisa Cohen, Diyaree Nath, Zara Habib, Xufei Guo, Igor Galynker, Raffaella Calati

**Affiliations:** 1 Department of Psychiatry, Mount Sinai Beth Israel, New York, New York, USA; 2 Department of Psychology, University of Milan-Bicocca, 20126 Milan, Italy; 3 Department of Adult Psychiatry, Nîmes University Hospital, 30029 Nîmes, France

**Keywords:** Suicide, Personality, Suicide Crisis Syndrome, Suicide Crisis Inventory

## Abstract

**Background:**

Personality traits have been associated with long-term suicide risk but their relationship with short-term risk is still unknown. Therefore, to address this gap, we explored the moderating effect of personality traits on the relationship between the Suicide Crisis Syndrome (SCS) and short-term suicidal behaviors (SB).

**Sampling and Methods:**

Adult participants (N = 459) were administered the Suicide Crisis Inventory (SCI), a validated self-report questionnaire designed to measure the intensity of the Suicidal Crisis Syndrome, the Big Five Inventory for personality traits, and the Columbia Suicide Severity Rating Scale for SB at intake and at a 1-month follow-up. The PROCESS macro in SPSS was used to test the moderation model. Covariates hypothesized to influence the results were added: age, gender, ethnicity, years of education, and depressive symptomatology on the Beck Depression Inventory. This study was a secondary analysis drawn from a larger study on the SCS.

**Results:**

SCI total score had a significant positive relationship with SB at the 1-month follow-up for patients with lower levels of extraversion, agreeableness, conscientiousness, and openness, respectively. Hence, these four traits were protective against SB. There was an association between SCI and SB for patients with high levels of neuroticism at the 1-month follow-up.

**Conclusions:**

High levels of neuroticism served as a risk factor, whereas high levels of the other Big Five traits were protective factors against short-term SB in the context of elevated SCS symptoms. Thus, personality traits play a role in moderating the relationship between the SCS and imminent SB.

## Introduction

Suicide claims over 700,000 lives in the world on a yearly basis [1]. A major public health concern, suicide is predicted to become an even greater burden in the upcoming decades [1,2]. In light of these facts, identifying risk factors for suicidal behaviors (SBs) in order to facilitate early intervention and healthcare policies for those at risk remains the primary focus of suicide prevention research. While long-term risk factors such as a patient’s demographic and familial characteristics, psychological traits, and stressful life events have been well-described [2,3], the risk factors for imminent suicide have not yet been clearly established [4–6].

Two long-term risk factors—a history of mental illness and past suicide attempts—have traditionally been identified as the best predictors for eventual SB, resulting in grouping of those having one, the other, or both into a universally accepted high suicide risk group [7,8]. However, recent studies of suicide prediction models based on these and other factors related to chronic mental illness and suicidal ideation (SI) revealed these models to be weak and inaccurate predictors of SB [7,8]. In fact, the low clinical utility of these models has not changed over the last 50 years [7,8].

The suicide crisis syndrome (SCS), a recently described predictor of *imminent* suicide risk, complements the traditional suicide risk predictor models by focusing on the acute presuicidal mental state [9–11]. The SCS is an acute state of cognitive and affective dysregulation that can develop within hours or days leading up to SB. The defining characteristics of the SCS are an intolerable state of frantic hopelessness (or entrapment) accompanied by loss of cognitive control, over-arousal, and social withdrawal [11–15]. Although the SCS has been shown to be a meaningful predictor of imminent SB in diverse clinical settings, additional work is necessary to further understand the syndrome and optimize its clinical use.

Personality traits are described as complex phenotypes, determined by the environment, individual genes, and gene–gene and gene–environment interactions [16]. Some personality features, including those identified within the Big Five framework, have been linked to SB risk [16, 17]. The Big Five personality traits are a widely accepted personality model comprising five broad domains: extraversion, agreeableness, conscientiousness, openness, and neuroticism [18]. Extraversion is the tendency to be enthusiastic, assertive, and outgoing; agreeableness represents warmth, trustworthiness, and reliability; conscientiousness is the tendency to display competence, self-discipline, and hard work; openness refers to the inclination toward varied experiences, esthetics, and creativity; and neuroticism is the tendency to experience emotional distress and instability [18].

Retrospective studies have shown high neuroticism to be associated with increased SI, attempts, and deaths, whereas high extraversion, conscientiousness and agreeableness are associated with lower SI, attempts, and deaths, suggesting that certain traits should be screened for when assessing SB risk [16,19,20]. The combination of long-term risk factors, which include personality traits, childhood maltreatment/trauma [21], and other psychological traits such as perfectionism [22], has been shown to predict to a life narrative characterized by alienated and debased self-perception, which in turn can lead to the emergence of the SCS, which then may lead to SB [23–25]. Thus, understanding how the potential interaction of personality traits and the SCS may help the identification of patient subgroups in need for early suicide prevention treatments [16,26]. Although personality traits are more amenable to modification relatively early in life, even interventions performed in adulthood could have long-lasting effects to potentially decrease the risk of SB [16,27,28].

Therefore, the purpose of the study is to explore the moderating effect of personality traits on the relationship between the SCS and short-term SB. We hypothesize that high neuroticism will serve as a risk factor in individuals with elevated SCS symptoms, followed by an escalated incidence of SB, whereas high levels of extraversion, agreeableness, conscientiousness, and openness would perform as protective factors, associated with a lesser incidence of SB.

## Methods

### Participants

The study sample consisted of 672 psychiatric outpatient participants recruited for a larger study at a network of New York City hospitals. This study was initiated in November of 2016 and closed recruitment in March of 2020 (at the beginning of the Covid-19 pandemic). The larger study aimed to validate a novel instrument known as the Multi-Informant Assessment of Risk for Imminent Suicide (MARIS) among psychiatric inpatient and outpatient participants. The current study was restricted to outpatient participants; however, the inclusion and exclusion criteria for outpatients in both the larger study and the current analyses were identical.

The inclusion criteria required participants to have adequate literacy and cognitive capacity to provide informed consent and to read and answer questionnaires, as well as to be fluent in English, domiciled, and at least 18 years of age. To minimize any potential treatment effect, recruitment was limited to individuals initiating treatment with a new psychiatric provider at the Mount Sinai Health System. Patients with cognitive impairment, severe psychotic symptoms, intellectual disability, or who were undomiciled (due to difficulties with obtaining follow-up data) were excluded from the study.

### Measures

Demographic information, which included age, gender, ethnicity, and years of education, was recorded and a psychological test battery assessing personality traits (Big Five Inventory, BFI), depressive symptoms (Beck Depression Inventory, BDI), suicidal thoughts and behaviors (Columbia-Suicide Severity Rating Scale, C-SSRS), and the SCS (Suicide Crisis Inventory, SCI).

#### Big Five Inventory

The BFI is a 44-item self-report inventory that measures the Big Five personality dimensions, namely Extraversion (8 items), Agreeableness (9 items), Conscientiousness (9 items), Neuroticism (8 items), and Openness (10 items) [29]. The respondents rate the items on a five-point scale (from “1 = disagree strongly” to “5 = agree strongly”). John and Srivastava reported Cronbach’s *α* from 0.75 to 0.80 for the reliability of the subscales and 3-month test–retest reliability from 0.80 to 0.90 [30]. In this dataset, the BFI demonstrated a standard level of internal consistency for four of the subscales (Cronbach’s *α* = 0.799 for Extraversion, Cronbach’s *α* = 0.751 for Agreeableness, Cronbach’s *α* = 0.794 for Conscientiousness, and Cronbach’s *α* = 0.828 for Neuroticism) with a lower alpha for the Openness subscale (Cronbach’s *α* = 0.672).

#### Beck Depression Inventory

The BDI is a widely used self-report scale containing 21 groups of statements to assess severity of depression [31]. Respondents choose one or more statements in each group (ranging from 0 to 3) and the highest number represents their score. Four items in the BDI (items 4, 11, 12, and 16) were excluded from the calculation of the BDI total score due to redundant items in the SCI. With the exclusion of these four items, the BDI demonstrated excellent internal validity (Cronbach’s *α* = 0.896).

#### Columbia-Suicide Severity Rating Scale

The C-SSRS is a semistructured interview administered by trained research assistants that assesses the presence and severity of suicidal thoughts and behaviors (STB). The intensity of SI was measured using a five-point scale (from “1 = Wish to be Dead” to “5 = ‘Active SI with Specific Plan and Intent’”). For the three types of SB—actual suicide attempt, interrupted attempt, and aborted attempt—scores were calculated on a binary scale (“0” indicating no SB, and “1” indicating the presence of at least one type of SB). To measure STB, a composite score was calculated on a scale of 1–9, reflecting the most severe level of STB in the specified time frame, with 1–5 indicating SI and 6–9 indicating preparations for a suicide attempt (6), or an aborted (7), interrupted (8), or actual suicide attempt (9). Although reliability coefficients were not calculated for the C-SSRS, the research team met weekly to review each C-SSRS and finalize scoring by consensus.

#### Suicide Crisis Inventory

The SCI is a 49-item self-report questionnaire that measures the intensity of the SCS [11]. Designed as an acute state measure, the SCI asks respondents to rate the intensity of each symptom on a 5-point scale (from “0 = not at all” to “4 = extremely”) based on their feelings over the past few days. In the current study, the SCI demonstrated excellent internal consistency (Cronbach’s *α* = 0.978) in adult psychiatric patients (*N* = 201).

### Procedure

The study participants were recruited from psychiatric outpatient services in four New York City hospitals in the Mount Sinai Health System. The clinicians, who were psychiatric residents working in those settings, referred potential participants after assessing the patients’ eligibility as per the inclusion criteria. Compensation was provided for the clinicians’ time for each patient referred. Interested patients were contacted within 2 weeks of the referral. All participants were provided informed consent and demographic information at the beginning of the study. Data on participants’ psychiatric diagnoses were drawn from the medical record. Trained research assistants administered a psychological test battery to each participant in-person. One-month post initial assessment, a follow-up assessment was administered in-person or via phone call. Participants were reimbursed $50 for completing the initial assessment and $25 for completing the 1-month follow-up assessment.

### Data analysis

Data analyses were conducted with SPSS version 25. Nonparametric analogs for parametric tests calculated the descriptive and bivariate analyses. The Mann–Whitney *U* test and Spearman’s correlations were used for univariate analyses involving the measures. Chi-squared statistics and ANCOVA analyses were used to compare groups with and without SB over the lifetime (from initial assessment), during the last 3-months (prior to initial assessment), and in the 1-month after initial assessment (1-month follow-up). For lifetime SB, ANCOVA controlled for age, years of education, and race; for SB during the past 3-months, ANCOVA controlled for age; and for SB at 1-month follow-up, no demographic variable needed to be controlled (for the covariates with significant differences between groups, see Supplementary Tables S3.1–S3.3, respectively). Logistic regression was used to test the hypothesized moderation model, that is, whether the Big Five personality traits moderate the effects of SCI on SB at 1-month follow-up. Logistic regression was conducted using the moderation model in PROCESS macro in SPSS. SCI and BFI were centered around their means before computing the interaction term, and all terms were entered into the model together. The Johnson–Neyman Procedure was used to probe the interaction: simple effects coefficients were calculated on three values of each personal trait: 1 SD below the mean, mean, and 1 SD above the mean.

## Results

### Descriptive statistics

A total of 672 outpatient participants completed the intake. Among them, 459 patients (68.3%) completed the 1-month follow-up, and 213 patients (31.7%) did not. This level of attrition was not unexpected, as other studies conducted in urban hospitals providing care to underserved low-income population have produced similar follow-up statistics [32]. Although the study did not tabulate the participant’s reasons for not completing the follow-up assessment, typical reasons include loss of interest, discomfort with disclosing suicidality, and failure to respond sufficiently.

Table 1 shows the demographic information for the outpatient sample at intake and at 1-month follow-up. There were no differences between the groups’ gender, ethnicity, race, or marital status. However, the group that completed the 1-month follow-up had significantly higher age (*Z* = 2.19, *p* = 0.03) and years of education (*Z* = 2.71, *p* = 0.007) than the group that did not.

**Table 1.** Demographics for whole outpatient sample and group differences for 1-month follow-up dropouts and completers.

Variable name	Total outpatient participants at intake (*N* = 672) *N* (%) or Mean [SD]	Completers at 1-month follow-up (*N* = 459) *N* (%) or Mean [SD]	Dropouts at 1-month follow-up (*N* = 213) *N* (%) or Mean [SD]	Group differences *Z*-value (*p*) or Chi-square (*p*)
Age	38.69 [14.27]	39.47 [14.25]	37.01 [14.19]	2.19* (0.03)
Years of education	14.23 [3.06]	14.44 [3.22]	13.79 [2.63]	2.71** (0.007)
Gender				1.60 (0.45)
Male	207 (30.8)	141 (30.7)	66 (31.0)	
Female	448 (66.7)	304 (66.2)	144 (67.6)	
Other	17 (2.5)	14 (3.1)	3 (1.4)	
Ethnicity				0.26 (0.60)
Hispanic/Latino	238 (35.4)	160 (34.9)	78 (36.6)	
Not Hispanic/Latino	428 (63.7)	296 (64.5)	132 (62.0)	
Race				5.0 (0.17)
Asian	43 (6.4)	36 (7.8)	7 (3.3)	
Black	163 (24.3)	110 (24.0)	53 (24.9)	
White	249 (37.1)	167 (46.4)	82 (38.5)	
Other	210 (31.3)	142 (30.9)	68 (31.9)	
Marital status				1.37 (0.85)
Never married	473 (70.4)	318 (69.3)	155 (72.8)	
Married	57 (8.5)	42 (9.2)	15 (7.0)	
Separated	32 (4.8)	23 (5.0)	9 (4.2)	
Divorced	95 (14.1)	66 (14.4)	29 (13.6)	
Widowed	14 (2.1)	9 (2.0)	5 (2.3)	

* *p* < 0.05;

** *p* < 0.01.

### Association between the clinical variables

As shown in Table 2, for the patients who completed the 1-month follow-up (*N* = 459), SCI total score was significantly positively associated with BDI total score (minus items 4, 11, 12, and 16) (*ρ* = 0.69, *p* < 0.001), SI in lifetime (*ρ* = 0.23, *p* < 0.001), SI in 1-month prior to initial assessment (*ρ* = 0.41, *p* < 0.001), and SI at 1-month follow-up (*ρ* = 0.30, *p* < 0.001). SCI was also positively associated with STB in lifetime (*ρ* = 0.21, *p* < 0.001), and STB at 1-month follow-up (*ρ* = 0.31, *p* < 0.001). For BFI subscale scores, SCI score was significantly related to each of the traits extraversion, agreeableness, conscientiousness, neuroticism, and openness (*ρ* = −0.30, *ρ* = −0.12, *ρ* = −0.33, *ρ* = −0.17, and *p* = 0.44, respectively; all of them had *p* < 0.001).

**Table 2.** Continuous clinical variables and their association with SCI total score (*N* = 459).

Variable name	Mean [SD]	Correlation coefficient with SCI total score (*ρ*)^a^
Extraversion	23.53 [6.98]	−0.30** (<0.001)
Agreeableness	32.56 [6.17]	−0.12** (<0.001)
Conscientiousness	29.70 [6.98]	−0.33** (<0.001)
Neuroticism	37.33 [7.08]	−0.17** (<0.001)
Openness	29.61 [6.48]	0.44** (<0.001)
BDI total score (minus items 4, 11, 12, and 16)	17.38 [10.06]	0.69** (<0.001)
SI lifetime	3.38 [1.89]	0.23** (<0.001)
SI last 1-month	1.31 [1.66]	0.41** (<0.001)
SI at 1-month follow-up	0.90 [1.37]	0.30** (<0.001)
STB lifetime	5.17 [3.53]	0.21** (<0.001)
STB at 1-month follow-up	1.00 [1.66]	0.31** (<0.001)

Abbreviations: BDI, Beck Depression Inventory; SCI, Suicide Crisis Inventory; SI, suicidal ideation; STB, suicidal thoughts and behaviors.

** *p* < 0.01.

a *ρ* values are Spearman’s *ρ.*

Table 3 compares SCI total scores between patients with past SB and those without. Patients who reported lifetime SB at the intake had higher SCI scores than patients who did not (*Z* = 2.90, *p* = 0.004), even after controlling for demographic covariates (*F* = 7.64, *p* = 0.006). Patients who reported SB in the last 3-month prior to the intake also had slightly, but not significantly, higher SCI scores than patients who did not (*Z* = 1.83, *p* = 0.068). However, after controlling for age and years of education, the two groups had no difference in SCI total score (*F* = 3.29, *p* = 0.07). Finally, patients who reported SB at the 1-month follow-up had significantly higher SCI scores than the patients who did not (*Z* = 3.11, *p* = 0.002).

**Table 3.** Binary clinical variables and their group differences on SCI total score (*N* = 459).

Variable name	*N* (%)	Mean [SD]	Mann–Whitney *U* test *Z*-value (*p*)	ANCOVA^a^ *F*-value (*p*)
SB lifetime			2.90** (0.004)	7.64** (0.006)
Yes	194 (42.3)	89.80 [48.56]		
No	238 (51.9)	76.39 [45.57]		
SB last 3-month			1.83 (0.068)	3.26 (0.07)
Yes	21 (4.6)	103.43 [55.94]		
No	411 (89.5)	81.34 [46.7]		
SB at 1-month follow-up			3.11** (0.002)	11.29** (0.001)
Yes	8 (1.7)	138.13 [52.79]		
No	451 (98.3)	82.19 [46.58]		

Abbreviations: SB, suicidal behavior; SCI, Suicide Crisis Inventory.

** *p* < 0.01.

a For all the binary outcome variables, ANCOVA controlled for the covariates that significantly differed between completers and drop out groups. For details, see section “Methods” and Supplementary Materials.

### Moderation analysis

Table 4 presents the parameters estimated using five moderation analyses, one for each personality trait. According to this table, all Big Five personality traits moderated the relationship between SCI and SB at the 1-month follow-up even after controlling for age, gender (female), ethnicity, and years of education.

**Table 4.** Moderation models.

	*X* = Extraversion		*X* = Agreeableness		*X* = Conscientiousness		*X* = Neuroticism		*X* = Openness	
	Coefficient		Coefficient		Coefficient		Coefficient		Coefficient	
Predictor	(SE)	*p*	(SE)	*p*	(SE)	*p*	(SE)	*p*	(SE)	*p*
Intercept	−7.486 (3.317)	0.024	−5.53 (2.717)	0.042	−5.313 (2.736)	0.052	−4.497 (2.597)	0.083	−4.449 (2.91)	0.126
SCI	0.048 (0.022)	0.026	0.034 (0.018)	0.057	0.031 (0.017)	0.076	0.022 (0.015)	0.152	0.035 (0.018)	0.056
*X*	0.261 (0.099)	0.008	0.238 (0.107)	0.027	0.166 (0.086)	0.054	−0.055 (0.087)	0.529	0.23 (0.117)	0.05
*X* × SCI	−0.004 (0.001)	0.003	−0.004 (0.002)	0.01	−0.004 (0.001)	0.017	0.002 (0.001)	0.03	−0.004 (0.002)	0.012
Age	−0.078 (0.044)	0.078	−0.078 (0.040)	0.051	−0.088 (0.044)	0.046	−0.074 (0.041)	0.072	−0.081 (0.044)	0.066
Male	−1.174 (1.213)	0.333	−0.954 (1.144)	0.405	−0.868 (1.147)	0.449	−1.028 (1.165)	0.378	−1.262 (1.229)	0.305
Female										
Ethnicity	0.576 (0.935)	0.538	0.381 (0.871)	0.662	0.517 (0.855)	0.546	0.427 (0.832)	0.608	0.586 (0.89)	0.51
Education	0.138 (0.175)	0.430	0.082 (0.143)	0.567	0.07 (0.15)	0.641	0.049 (0.142)	0.728	0.039 (0.169)	0.818
*BDI*	0.066 (0.059)	0.262	0.064 (0.056)	0.249	0.075 (0.059)	0.204	0.053 (0.057)	0.354	0.039 (0.058)	0.501
Model *R* ^2^ (McFadden)	0.324	<0.001	0.324	0.001	0.313	0.002	0.286	0.004	0.33	0.001

*Note: Logistic regression model coefficients for suicidal behavior at 1-month follow-up. SE in parentheses.*

Abbreviations: BDI, Beck Depression Inventory; SCI, Suicide Crisis Inventory.

The logistic regression results indicate that the interaction between SCI and extraversion was significant in predicting SB at the 1-month follow-up (*b* = −0.004, *SE* = 0.001, *p* = 0.003). Specifically, SCI exhibited a positive association with SB at low (*b* = 0.078,  *SE* = 0.029, *OR* = e0.078 = 1.081, *p* = 0.006) and mean (*b* = 0.05, *SE* = 0.021, *OR* = e0.05 = 1.051, *p* = 0.021) levels of extraversion, but not a high (*b* = 0.021,  *SE* = 0.017, *OR* = e0.021 = 1.021, *p* = 0.200) level of extraversion.

The interaction between SCI and agreeableness also resulted in a significant prediction of SB at the 1-month follow-up (*b* = −0.004, *SE* = 0.002, *p* = 0.01). As with extraversion, SCI was positively associated with SB at a low (*b* = .0059,  *SE* = 0.024, *OR* = 1.061, *p* = 0.015) level of agreeableness. However, there was no difference between SCI and SB at the mean (*b* = 0.035, *SE* = 0.018, *OR* = 1.036, *p* = 0.051) and high (*b* = 0.010, *SE* = 0.015, *OR* = 1.010, *p* = 0.497) levels of agreeableness.

Similarly, the interaction between SCI and conscientiousness was also significant in predicting SB at the 1-month follow-up (*b* = −0.004, *SE* = 0.001, *p* = 0.017). SCI was positively associated with SB at a low (*b* = 0.057, *SE* = 0.024, *OR* = 1.059, *p* = 0.019) level of conscientiousness, but there was no apparent difference between SCI and SB at the mean (*b* = 0.032, *SE* = 0.017, *OR* = 1.033, *p* = 0.062) and high (*b* = 0.007, *SE* = 0.015, *OR* = 1.007, *p* = 0.625) levels of conscientiousness.

The interaction of SCI with neuroticism was again significant in predicting SB at the 1-month follow-up (*b* = 0.002,  *SE* = 0.001, *p* = 0.03). However, unlike the interaction of SCI with the other personality traits, there was no difference between SCI and SB at low (*b* = 0.008, *SE* = 0.016, *OR* = 1.083, *p* = 0.615) and mean (*b* = 0.023, *SE* = 0.015, *OR* = 1.023, *p* = 0.132) levels of neuroticism. Instead, there was a positive association between SCI and SB at a high (*b* = 0.038, *SE* = 0.018, *OR* = 1.039, *p* = 0.031) level of neuroticism.

Finally, the interaction between SCI and openness was also significant in predicting SB at the 1-month follow-up (*b* = −0.004, *SE* = 0.002, *p* = 0.012). SCI was positively associated with SB at a low (*b* = 0.064, *SE* = 0.026, *OR* = 1.066, *p* = 0.015) level of openness, but there was no difference between SCI and SB at mean (*b* = 0.035, *SE* = 0.018, *OR* = 1.036, *p* = 0.054) and high (*b* = 0.007, *SE* = 0.015, *OR* = 1.007, *p* = 0.654) levels of openness.

Thus, the overall results demonstrated that low but not medium or higher levels of extraversion, agreeableness, conscientiousness, and openness were risk factors for SB at the 1-month follow-up with participants exhibiting SCS whereas SCI predicted SB only at high levels of neuroticism.

## Discussion

Our findings indicate that the Big Five personality traits have significantly moderated the relationship between individuals experiencing the SCS and short-term risk of SB. In particular, the results showed that high but not medium or low levels of neuroticism were significantly associated with SB among individuals with SCS, thus demonstrating that high levels of neuroticism serve as a risk factor. In contrast, high levels of extraversion, agreeableness, conscientiousness, and openness were all found to serve as protective factors against SB among individuals exhibiting SCS. The overall findings, therefore, support our initial hypothesis that high levels of neuroticism potentially identify those at higher risk for SB, whereas high levels of extraversion, agreeableness, conscientiousness, and openness perform as protective factors, identifying those at lower risk among individuals exhibiting SCS.

The present findings resemble previous results regarding the role of personality traits in relation to SB [16, 17]. The literature shows a robust association between neuroticism as a personality trait and many other indices of psychopathology [33,34], even physiological indices of inflammation [35]. However, unlike previous studies that only concerned the relationship of personality traits with long-term SB, the present results establish neuroticism as a moderating influence that magnifies the chances of imminent SB in individuals with SCS. A potential explanation for this moderating effect is the shared characteristics between SCS and neuroticism. Neuroticism is characterized by emotional instability, anxiety, impulsivity, and distress, all of which could be present in the acute state of SCS [18]. The defining characteristic of SCS, however, is a crescendo onset of entrapment/frantic hopelessness, with affective dysregulation loss of cognitive control, over-arousal, intense distress, and social withdrawal [9–11]. High levels of trait neuroticism might exacerbate the evolving cognitive and affective dysregulation of an individual with SCS, increasing the likelihood of suicide being perceived as the only means of escaping unbearable emotional pain and making SB more likely. However, further research is necessary to identify the specific facets of personality traits involved in suicide risk and whether the increased risk is associated with certain personality traits per se or with a more global risk dimension that might include genetic susceptibility and/or gene–environment interactions [36,37].

Our other significant finding is the protective function of personality traits other than neuroticism. High levels of extraversion, agreeableness, conscientiousness, and openness conferred decreased probability of imminent SB in individuals with SCS. One possible explanation of this result is that the positive features of the traits that may reduce the impact of SCS symptoms. Conscientiousness has been shown to be protective [35] as has Agreeableness [38]. Conscientiousness and Agreeableness encompass characteristics that are considered positive for the self [18,39–41]. In particular, the characteristics of creativity, enthusiasm, warmth, trustworthiness, and competence may have an uplifting effect on an individual, influence an optimistic mindset and provide the capability to cope with negative emotions [18,39–41]. The sense of optimism and the ability to cope may then alleviate the cognitive symptoms of SCS that make one perceive life as a dead-ended sequence of events, thereby reducing the risk of imminent SB.

Research on the effect of Openness is more mixed, as it has been shown to associate with sensation seeking and impulsive aggression [38] as well as the personality trait of psychoticism [42]. In the context of the current study, openness might have counteracted the overly rigid thought process characteristic of the SCS. Similarly, extraversion has been associated with both positive and negative psychological features. Extraversion has been related to externalizing disorders, such as bipolar disorder, plus antagonism and disinhibition [43], although it is seen less frequently in internalizing disorders, such as depression and anxiety. In the same Watson et al. [43] study, extraversion was broken down into 4 subscales, which had differential relations to SI. Positive emotion and sociability subscales were negatively associated with SI, whereas assertiveness and experience seeking were positively associated with SI. It is likely that in this population, extraversion’s protective effects against depression and anxiety are specifically helpful with regards to moderating the effect of the SCS symptoms on near-term suicidal risk.

Another potential explanation for the protective function of extraversion, agreeableness, conscientiousness, and openness is their role in the quality and maintenance of interpersonal relationships. A prominent component of the SCS is social withdrawal, which comprises withdrawal from social activities and reduced and/or evasive communication with others [9–11]. Research has indicated that social withdrawal is one of the strongest predictors of SB [44,45] and that social and family support may be protective against SB among various populations [7,45]. Therefore, since high levels of extraversion, agreeableness, conscientiousness, and openness are congruent with interpersonal relationships, these traits may play a role in reducing the social withdrawal factor of SCS. Further research is necessary to distinguish these or potential other explanations.

### Limitations

The results of this study should be considered in the context of several limitations: firstly, the main limitation of this study related to the size and sample of the participants. The sample of participants with SB at 1-month follow-up only comprised 8 participants, which is a relatively small number. In addition, the participants who completed a 1-month follow-up comprised a relatively homogenous sample of older and educated psychiatric outpatient participants, suggesting some degree of self-selection among the study’s completers. These factors limit the generalizability of the findings. Further studies should replicate the present one with a larger sample size with a more diverse group of participants in order to support the current findings. Secondly, all of the measures were administered via self-report which may have led to an overestimation or underestimation of SCS and personality traits due to a potential misinterpretation of the question and/or a lack of disclosure on the part of the participants. In addition, the 1-month follow-up was conducted via phone wherein the researcher read out the questions and recorded the participant’s responses, which could also have led to misinterpretation. Moreover, the Big Five personality traits were the only measurement for certain traits in the study, limiting our general understanding of personality traits and disorders with SB. Although outside the scope of this study, assessing other personality traits as well as other mental disorders in relation to SB might assist in expanding the knowledge of risk factors and protective factors. Furthermore, the study did not consider comorbidity of the Big Five with DSM-5 personality disorders or other personality traits associated with SB, which should be further explored. Another limitation was the limited number of assessment points in the study. Therefore, the study was not able follow the progress and change of the relationship between SCS and short-term SB over a longer period of time. Finally, the BDI total score was calculated excluding four items (items 4, 11, 12, and 16) due to redundancy with the SCI, and Cronbach’s alpha was relatively low for the Openness scale. These factors could have limited the validity, reliability, and generalizability of these scales and findings.

### Theoretical implications and practical applications

Despite the efforts of numerous researchers and clinical groups, suicide continues to be difficult to predict and suicide rates across the United States continue to rise [1]. It is essential for us to expand our knowledge and understanding of imminent risk in order to better predict and diagnose presuicidal mental states [13]. Our results describing the relationship between the Big Five personality traits and near-term SBs are qualitatively similar to the previous work on the relations between personality traits and long-term SBs. This suggests that suicide risk conferred by personality traits transcends the risk acuity and, with further research could be incorporated in suicide risk assessment. Focusing on specific personality traits, such as high neuroticism, to systematically identify those who may be at increased imminent risk when under stress may improve clinical decision-making [13]. Hence, the assessment of personality traits, using for example the BFI, may be useful for the identification of more vulnerable patients. When other risk factors are present, the evaluation of personality-related protective and risk factors may help to build a more complete picture of the patient and to choose the intervention. However, to be able to implement the research in clinical practice, this study will need to be replicated with a higher number of suicidal outcomes.

Notwithstanding its limitations, the present study underscores the importance of personality traits in relation to SB. The Big Five personality traits were found to play a role in moderating the relationship between SCS and imminent SB, with neuroticism serving as a potential risk factor and the other traits serving as protective factors. These findings, supported by further research, might improve clinicians’ ability to identify those at immediate risk of suicide, thereby allowing early intervention and treatment to reduce the risk of suicide attempt.

## Data Availability

All relevant data are within the paper, and data is available upon the request to the authors. For supplementary material accompanying this paper, visit https://urldefense.proofpoint.com/v2/url?u=http-3A__cambridge.org_EPA&d=DwIGaQ&c=shNJtf5dKgNcPZ6Yh64b-A&r=B0jucilxA9aUc45oTwTECX-_MZBQsG3ltRayKN9Gd70&m=zkAMlNiJHJrQKfzJqgbt0_szPBo2G7pFUf_GBXyMxgU&s=1Rr2p1fUnsOGFNERjaE2gjY-sHot0Ym2xfid5TiqUHc&e=.

## Supplementary Materials

To view supplementary material for this article, please visit http://dx.doi.org/10.1192/j.eurpsy.2021.2235. click here to view supplementary material
