# Supplementary material for: The association between the suicide crisis syndrome and suicidal behaviors: The moderating role of personality traits
Source: Eur Psychiatry. 2021 Oct 13;64(1):e63. doi: 10.1192/j.eurpsy.2021.2235 (PMC8581701; doi:10.1192/j.eurpsy.2021.2235)
Supplement: Supplementary file 1 [file S0924933821022355sup001.docx]

**Supplementary Materials**

**Table S3.1** Patient Characteristics for Groups with/without SB at Lifetime (N=432)

| Variable name | With suicidal behavior (N=194)  N (%) or Mean [SD] | Without suicidal behavior (N=238)  N (%) or Mean [SD] | Z-value/Chi-square value  [p-value] |
| --- | --- | --- | --- |
| Age | 37.66 [13.76] | 41.26 [14.45] | 2.58* [0.01] |
| Years of education | 14.01 [3.02] | 14.80 [3.25] | 2.44* [0.02] |
| Gender |  |  | 1.94 [0.38] |
| Male | 54 (27.8) | 80 (33.6) |  |
| Female | 135 (73.4) | 154 (64.7) |  |
| Other | 5 (2.6) | 4 (1.7) |  |
| Ethnicity |  |  | 0.01 [0.93] |
| Hispanic/Latino | 67 (34.7) | 81 (34.3) |  |
| Not Hispanic/Latino | 126 (65.3) | 155 (65.7) |  |
| Race |  |  | 23.4** [<0.001] |
| Asian | 7 (3.6) | 27 (11.5) |  |
| Black | 66 (34.2) | 39 (16.6) |  |
| White | 64 (33.2) | 95 (40.4) |  |
| Other | 56 (20.0) | 74 (30.5) |  |
| Marital Status |  |  | 4.04 [0.40] |
| Never married | 140 (72.2) | 157 (66.2) |  |
| Married | 13 (6.7) | 28 (11.8) |  |
| Separated | 9 (4.6) | 12 (5.1) |  |
| Divorced | 29 (14.9) | 34 (14.3) |  |
| Widowed | 3 (1.5) | 6 (2.5) |  |

* p<0.05; ** p<0.01

**Table S3.2** Patient Characteristics for Groups with/without SB During the Last 3 Months (N=432)

| Variable name | With suicidal behavior (N=21)  N (%) or Mean [SD] | Without suicidal behavior (N=411)  N (%) or Mean [SD] | Z-value/Chi-square value  [p-value] |
| --- | --- | --- | --- |
| Age | 31.24 [12.93] | 40.07 [14.19] | 3.00** [0.003] |
| Years of education | 14.31 [4.17] | 14.45 [3.09] | 0.30 [0.77] |
| Gender |  |  | 0.88 [0.64] |
| Male | 7 (33.3) | 127 (30.9) |  |
| Female | 13 (61.9) | 276 (60.2) |  |
| Other | 1 (4.8) | 8 (6.3) |  |
| Ethnicity |  |  | 0.01 [0.91] |
| Hispanic/Latino | 7 (33.3) | 141 (34.6) |  |
| Not Hispanic/Latino | 14 (66.7) | 267 (65.4) |  |
| Race |  |  | 6.02 [0.11] |
| Asian | 0 (0) | 34 (8.3) |  |
| Black | 9 (45.0) | 98 (24.0) |  |
| White | 7 (35.0) | 152 (37.3) |  |
| Other | 4 (20.0) | 126 (30.9) |  |
| Marital Status |  |  | 1.02 [0.91] |
| Never married | 16 (76.2) | 281 (68.5) |  |
| Married | 2 (9.5) | 39 (9.5) |  |
| Separated | 1 (4.8) | 20 (4.9) |  |
| Divorced | 2 (9.5) | 61 (14.9) |  |
| Widowed | 0 (0) | 9 (2.2) |  |

* p<0.05; ** p<0.01

**Table S3.3** Patient Characteristics for Groups with/without SB at 1-month Follow-up (N=459)

| Variable name | With suicidal behavior (N=8)  N (%) or Mean [SD] | Without suicidal behavior (N=451)  N (%) or Mean [SD] | Z-value/Chi-square value  [p-value] |
| --- | --- | --- | --- |
| Age | 30.38 [10.73] | 39.63 [14.27] | 1.87 [0.06] |
| Years of education | 14.31 [2.84] | 14.44 [3.23] | 0.01 [0.99] |
| Gender |  |  | 1.68 [0.43] |
| Male | 1 (12.5) | 140 (31.0) |  |
| Female | 7 (87.5) | 297 (65.9) |  |
| Other | 0 (0) | 14 (3.1) |  |
| Ethnicity |  |  | 2.69 [0.14] |
| Hispanic/Latino | 5 (62.5) | 155 (34.4) |  |
| Not Hispanic/Latino | 3 (37.5) | 293 (65.0) |  |
| Race |  |  | 1.60 [0.66] |
| Asian | 0 (0) | 36 (8.0) |  |
| Black | 1 (12.5) | 109 (24.2) |  |
| White | 4 (50.0) | 163 (36.1) |  |
| Other | 3 (37.5) | 139 (30.8) |  |
| Marital Status |  |  | 5.37 [0.25] |
| Never married | 3 (37.5) | 315 (69.8) |  |
| Married | 1 (12.5) | 41 (9.1) |  |
| Separated | 1 (12.5) | 22 (4.9) |  |
| Divorced | 3 (37.5) | 63 (14.0) |  |
| Widowed | 0 (0) | 9 (2.0) |  |

* p<0.05; ** p<0.01
